# Severe reflux, sleep disturbances, and health-related quality of life after esophageal cancer surgery

**DOI:** 10.1007/s11764-020-00974-9

**Published:** 2021-01-27

**Authors:** Pernilla Lagergren, Asif Johar, Helen Rosenlund, Lars Arnberg, Lena Haglund, Eivind Ness-Jensen, Anna Schandl

**Affiliations:** 1 Surgical Care Science, Department of Molecular medicine and Surgery, Karolinska Institutet, Karolinska University Hospital, 171 76 Stockholm, Sweden; 2 Department of Surgery & Cancer, Imperial College London, London, UK; 3 Division of Nutrition and Dietetics, Department of Orthopaedics, Danderyd Hospital, Stockholm, Sweden; 4 Patient research partnership group, Surgical Care Science, Department of Molecular medicine and Surgery, Karolinska Institutet, Karolinska University Hospital, 171 76 Stockholm, Sweden; 5 HUNT Research Centre, Department of Public Health and Nursing, NTNU, Norwegian University of Science and Technology, Levanger, Norway; 6 Department of Medicine, Levanger Hospital, Nord-Trøndelag Hospital Trust, Levanger, Norway; 7 Department of Anaesthesiology and Intensive Care, Södersjukhuset, Stockholm, Sweden

**Keywords:** Oesophageal neoplasm, Insomnia, HRQL, Cancer survivorship

## Abstract

**Purpose:**

Esophagectomy for cancer is an extensive procedure often followed by severe complications. This study investigated whether patients with severe symptoms of reflux are more likely to have sleep disturbances and reduced health-related quality of life (HRQL) after esophagectomy.

**Methods:**

This Swedish nationwide prospective cohort study encompassed all patients who had undergone esophagectomy for cancer between 2013 and 2018. One year after surgery, the patients responded to three questionnaires on reflux (EORTC QLQOG25), sleep disturbances (KSQ), and HRQL (EORTC QLQ-C30). Multivariable logistic regression provided odds ratios (OR) with 95% confidence intervals (CI) for sleep disturbance/reduced HRQL between patients with and without reflux, adjusted for potential confounders.

**Results:**

Among 241 esophagectomy patients, 66 (27%) reported severe reflux. Patients with reflux had an increased risk of sleep disturbances (OR 2.3, 95% CI: 1.3–4.3) compared to patients without reflux. More specifically, these patients were more likely to suffer from poor sleep quality (OR 4.9, 95% CI: 1.9–12.4). Patients with reflux and sleep disturbances reported reductions in global quality of life, role function, emotional function, social function, and more symptoms in all scales, except for dyspnea.

**Conclusions:**

This study suggests that patients with severe symptoms of reflux after esophagectomy have an increased risk of sleep disturbances and poor sleep quality, which in turn are associated with reduced HRQL.

**Implications for Cancer Survivors:**

Alleviating reflux after oesophageal cancer surgery is important, since this common symptom might reduce HRQL and well-being.

## Introduction

Esophagectomy for cancer is an extensive procedure. A large part of the oesophagus with its tumor is removed and typically replaced with the stomach which is reconstructed into a tube [1]. The treatment is often followed by severe complications [2–4]. However, for patients without metastasis, the 1-year survival rate is approximately 80% [5]. The majority of these patients suffer from debilitating symptoms and reduction in health-related quality of life (HRQL) years after surgery [6–8]. Reflux often occurs after esophagectomy because the surgical procedure disrupts the normal antireflux barrier. The negative intrathoracic pressure and the intraabdominal pressure also promote reflux across the anastomosis [9]. This is often regarded as inevitable sequelae after surgery, but is nevertheless a considerable problem for the individual patient. A cardinal symptom of reflux is regurgitation of stomach content, which is particularly burdensome when the patient is in the supine position. Nightly regurgitations might contribute to sleep disturbances. Previous studies have indicated an association between gastroesophageal reflux disease and sleep problems [10, 11]. However, whether such an association exists for patients who have undergone oesophageal cancer surgery has not yet been clarified. Since reflux is a significant problem after esophagectomy, former oesophageal cancer patients suggested a potential study on this topic. Therefore, a nationwide prospective study was conducted, in collaboration with a research partner group consisting of former oesophageal cancer patients, hypothesizing that patients who have undergone esophagecomy for oesophageal cancer and suffer from severe reflux are more likely to have sleep disturbances and reduced HRQL.

## Methods

This was a nationwide, population-based, prospective cohort study covering almost all oesophageal cancer patients who underwent esophagectomy in Sweden between 2013 and 2018 and were alive 1 year postoperatively. Eligible patients were identified through collaboration with eight pathology departments in all Swedish hospitals where these operations were conducted. The cohort has been described in detail elsewhere [12]. The Regional Ethical Review Board in Stockholm, Sweden (diary number 2013/844–31/1), approved the project and all participants gave informed consent. A research partnership group consisting of former oesophageal cancer patients was involved as co-researchers in the study.

### Data collection

After a first contact by mail, 1 year after surgery, followed by a phone call for acceptance of participation, a research nurse visited the patients in their homes in order to guide them through the self-reported computer-based questionnaires. Clinical data were collected from medical records and included co-morbidities, tumor histology, site and stage, cancer treatment, and postoperative complications. Each medical record was reviewed by two researchers according to a predefined study protocol to ensure consistency and uniformity of the data collection. Cross-validation of randomly selected protocols was performed by an independent researcher. Data on patient characteristics were collected by linking the personal identity number assigned to each Swedish resident to national health data registries. Socio-demographic information was obtained by linkage to the Longitudinal Integration Database for Health Insurance and Labour Market, which holds registration since 1990 and is updated yearly [13]. Further information on comorbidities was obtained from the Swedish Patient Registry and the Swedish Cancer Registry [14]. The Swedish Register of the Total Population was used to retrieve mortality data. All these registries hold nearly 100% complete nationwide information [15, 16].

### Exposure

The study exposure was reflux, measured in the European Organisation for Research and Treatment of Cancer (EORTC) Quality of Life Questionnaire (QLQ) module for gastro-oesophageal symptoms (OG25) [17]. The QLQ-OG25 comprises six symptom scales (dysphagia, eating restrictions, reflux, odynophagia, pain, and discomfort and anxiety) and ten single items (eating in front of others, dry mouth, trouble with taste, body image, trouble swallowing saliva, choking when swallowing, coughing, trouble talking, weight loss, and hair loss). There are four response alternatives: “not at all,” “a little,” “quite a bit,” and “very much.” Reflux was identified in the questions “Have you had acid indigestion or heartburn?*”* and “Has acid or bile coming into your mouth been a problem?.” Patients who answered “quite a bit” or “very much” in either of the items were considered to have severe reflux.

All patients also responded to two study specific questions: “Do you sleep with an elevated head rest?” and “Do you use medication for reflux problems?,” both with “yes” and “no” as response alternatives. The responses were used to stratify the analyses according to symptom management strategies.

### Outcomes

The main outcome was sleep disturbances 1 year after surgery. Sleep and sleepiness were measured with the 18-item Karolinska Sleep Questionnaire (KSQ) in four dimensions (poor sleep quality, non-restorative sleep, daytime sleepiness, and obstructive breathing) [18]. It also provides an index of nocturnal insomnia symptoms. The dimension of sleep quality contains questions regarding premature awakenings, disturbed sleep, and repeated awakenings with difficulty going back to sleep. The dimension of non-restorative sleep consists of questions about difficulties waking up and not being well-rested on awakening. The sleepiness dimension includes questions regarding daytime sleepiness, such as the need to fight sleep to stay awake, and mental fatigue during the daytime. The dimension of obstructive breathing consists of questions about heavy snoring and cessation of breathing during sleep. In the KSQ, there are six response alternatives: “never,” “seldom/occasionally,” “sometimes/several times per month,” “often/1–2 times per week,” “most of the time/3–4 times per week,” and “always/5 times or more per week” and the participants were asked to consider their sleep for the past 3 months. The score for each dimension was calculated as the mean across items. The diagnosis of insomnia includes both nocturnal problems and daytime consequences. However, in the present study, only information on nocturnal problems was provided. Therefore, the insomnia index was based solely on two dimensions: sleep quality and non-restorative sleep. Each question in the index was dichotomized as 0 = “never”/“seldom/occasionally”/“sometimes/several times per month” and 1=“often/1-2 times per week”/“most of the time/ 3-4 times per week” to reflect poor sleep quality more than three times per week according to the Diagnostic and Statistical Manual of Mental Disorder-5 (DSM-V) criteria for insomnia [19]. Thereafter, the index was summed and again dichotomized between 0 (no symptoms) and 1 (one or more symptoms).

The secondary outcome was HRQL 1 year after esophagectomy. HRQL was assessed by the EORTC QLQ 30-item core questionnaire (QLQ-C30) [20]. The QLQ-C30 measures HRQL aspects with five multi-item functional scales (physical, role, cognitive, emotional and social), one global quality of life scale, three symptom scales (fatigue, pain, and nausea/vomiting), and six single-item measuring symptoms common among patients with cancer in general (dyspnoea, insomnia, appetite loss, constipation and diarrhea) and financial impact.

For the QLQ-C30, there are four response alternatives: “not at all,” “a little,” “quite a bit,” and “very much.” The only exception is the global quality of life scale, which has a seven-graded rating, ranging from 1 (“very poor”) to 7 (“excellent”). Responses to the functions and symptoms were further dichotomized into “no function reduction”/“no or minor symptoms” and “function reductions”/“symptomatic” in accordance with previous research [21, 22]. Patients who had at least one response of “quite a bit” or “very much” to any item within a scale were categorized as having “function reductions” or “symptoms.” Otherwise, patients were categorized as having “no function reductions” or “no or minor symptoms.” For the global quality of life scale, a response of 1 (very poor) and 2 (poor) were rated as having “reduced HRQL.” Otherwise, patients were rated as having “no HRQL reductions.”

### Confounders

Potential confounders were (1) age in years (continuous); (2) sex (male or female); (3) comorbidity (Charlson Comorbidity Index score: 0, 1, or ≥ 2); (4) psychiatric diagnosis (data were collected from the medical records and categorized as present or not present); (5) body mass index (BMI < 30 or ≥ 30) objectively measured by the research nurse; (6) smoking habits (self-reported data, categorized as former or current smoker and never smoker); (7) alcohol consumption (self-reported data, categorized as “never” = never been drinking alcohol and “ever” = have been drinking alcohol); (8) histology (adenocarcinoma or squamous cell carcinoma); (9) type of surgery (open, minimally invasive or hybrid) [23]; (10) tumor stage (0–I, II, or III–IV; (11) neoadjuvant therapy (yes or no); and (12) postoperative complications (Clavien Dindo: 0–II or III–IV) [24, 25].

### Statistical analyses

Patient characteristics were presented in numbers and percentages. Multivariable logistic regression was used to calculate odds ratios (OR) with 95% confidence intervals (CI) for sleep disturbance/reduced HRQL between patients with and without reflux. All analyses were adjusted for the twelve potential confounders. Patients with reflux were stratified in groups according to whether they used antireflux medication, slept with an elevated headrest, or had combined medication and elevation of the headrest. All data management and statistical analyses were conducted by a senior statistician (AJ) with expertise in HRQL analyses using SAS version 9.4 (Cary, NC).

## Results

### Patients

**Fig. 1:**
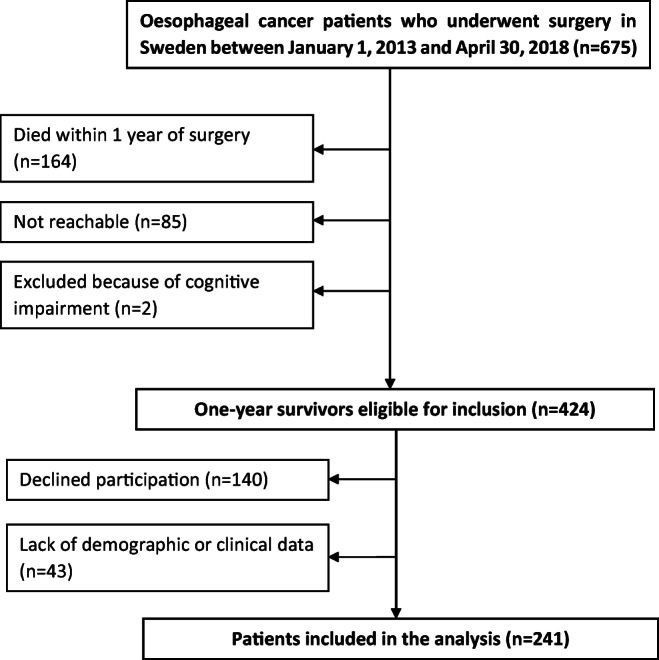
Flow chart of patient selection for inclusion

Between January 1, 2013, and April 30, 2018, 675 patients underwent oesophageal cancer surgery in Sweden. Of these, 511 (76%) survived for at least 1 year, 85 were not reachable, 2 excluded because of cognitive impairment, leaving 424 patients eligible for inclusion. Response rate was 67%, and complete data were present for 241 patients (Fig. 1).

The mean age was 66 ± 9 years and the majority were men (86%), with no comorbidities (47%) and adenocarcinoma (84%). Among these patients, 66 (27%) reported severe reflux (Table 1).

**Table 1:** Characteristics of patients with and without reflux 1 year after oesophageal cancer surgery

	All patients	No reflux	Reflux
Total number (%)	241 (100)	175 (73)	66 (27)
Age			
mean ± SD	66 ± 9	67 ± 9	65 ± 8
Sex			
Men	207 (86)	155 (89)	52 (79)
Women	34 (14)	20 (11)	14 (21)
Charlson comorbidity index score			
0	114 (47)	87 (50)	27 (41)
1	79 (33)	55 (31)	24 (36)
≥ 2	48 (20)	33 (19)	15 (23)
Psychiatric diagnosis			
No	117 (73)	173 (99)	65 (98)
Yes	64 (27)	2 (1)	1 (2)
Body mass index			
< 30	98 (41)	75 (43)	23 (35)
≥ 30	143 (59)	100 (57)	43 (65)
Smoking habits			
Current/former smoker	162 (67)	119 (68)	43 (65)
Never smoker	79 (33)	56 (32)	23 (35)
Alcohol drinking habits			
Ever	195 (81)	142 (81)	53 (80)
Never	46 (19)	33 (19)	13 (20)
Tumor histology			
Adenocarcinoma	202 (84)	147 (84)	55 (83)
Squamous cell carcinoma	39 (16)	28 (16)	11 (17)
Tumor stage			
0–I	80 (33)	57 (33)	23 (35)
II	82 (34)	59 (34)	23 (35)
III–IV	79 (33)	59 (34)	20 (30)
Type of surgery			
Open	92 (38)	67 (38)	25 (38)
Minimally invasive	75 (31)	54 (31)	21 (32)
Hybrid	74 (31)	54 (31)	20 (30)
Neoadjuvant therapy			
Yes	189 (78)	139 (79)	50 (76)
No	52 (22)	36 (21)	16 (24)
Clavien Dindo score (postoperative complications)			
0–II	147 (61)	103 (59)	44 (67)
III–IV	94 (39)	72 (41)	22 (33)

*SD* standard deviation

### Reflux and sleep disturbances

Esophagectomy patients with reflux were more likely to suffer from sleep disturbances (OR 2.3, 95% CI: 1.3–4.3), compared to patients without reflux (Table 2). More specifically, these patients were more likely to suffer from poor sleep quality, described as premature awakenings, disturbed sleep, and repeated awakenings (OR 4.9, 95% CI: 1.9–12.4) and experiencing non-restorative sleep (OR 2.8, 95% CI: 1.4–5.7), defined as difficulties waking up and not being well-rested on awakening. Patients who slept with an elevated headrest and used antireflux medications, indicating severe reflux, were more likely to suffer from non-restorative sleep (OR 4.8, 95% CI: 1.5–15.3) and daytime sleepiness (OR 5.6, 95% CI: 1.8–17.9).

**Table 2:** Associations between reflux in esophagectomy patients and sleep disturbances, presented as odds ratios (OR) with 95% confidence intervals (CI)

	Reflux		Reflux + elevated headrest		Reflux + medications		Reflux + elevated headrest + medications	
	Numbers	OR (95% CI)	Numbers	OR (95% CI)	Numbers	OR (95% CI)	Numbers	OR (95% CI)
Nocturnal insomnia (index)	41	2.3 (1.3–4.3)	11	2.7 (0.9–8.5)	36	2.2 (1.2–4.3)	10	2.8 (0.8–9.7)
Poor sleep quality	17	4.9 (1.9–12.4)	3	1.3 (0.3–6.3)	15	4.3 (1.7–11.1)	3	1.3 (0.3–6.5)
Non-restorative sleep	25	2.8 (1.4–5.7)	8	4.1 (1,3–12.7)	23	2.8 (1.4–5.8)	8	4.8 (1.5–15.3)
Daytime sleepiness	18	1.8 (0.9–3.8)	8	4.4 (1.4–13.1)	17	2.1 (1.0–4.3)	8	5.6 (1.8–17.9)
Obstructive breathing	8	1.0 (0.4–2.7)	1	0.8 (0.3–7.2)	7	1.1 (0.4–3.2)	1	0.8 (0.1–7.6)

Adjusted for age, sex, comorbidity, psychiatric diagnosis, body mass index, smoking habits, alcohol intake, type of surgery, tumor stage, neoadjuvant therapy, and postoperative complications

### Reflux with sleep disturbances and HRQL

Esophagectomy patients with reflux and sleep disturbances had increased risk of reduced global quality of life (OR 3.0, 95% CI: 1.0–9.0), role function (OR 3.1, 95% CI: 1.3–7.0), emotional function (OR 7.6, 95% CI: 3.2–18.4), and social function (OR 6.0, 95% CI: 2.7–13.3) (Table 3). These patients were also more likely to suffer from more symptoms in all scales and items in QLQ-C30, apart from dyspnea. Particularly high scores were reported for fatigue (OR 3.5, 95% CI: 1.6–7.7), pain (OR 4.7, 95% CI: 2.1–10.8), constipation (OR 4.4, 95% CI: 1.2–16.3), and financial difficulties (OR 11.1, 95% CI: 3.4–36.5).

**Table 3:** Associations between sleep disturbances in esophagectomy patients with reflux and functional reductions and symptoms presented as odds ratios with 95% confidence intervals

Reflux		
Health-related quality of life aspects	No sleep disturbances (reference) (*n* = 106)	Sleep disturbances (*n* = 41)
EORTC QLQ-C30		
Global quality of life	1.0	3.0(1.0–9.0)
Functional scales		
Physical function	1.0	1.5 (0.7–3.4)
Role function	1.0	3.1 (1.3–7.0)
Cognitive function	1.0	2.4 (0.9–6.2)
Emotional function	1.0	7.6 (3.2–18.4)
Social function	1.0	6.0 (2.7–13.3)
Symptom scales		
Fatigue	1.0	3.5 (1.6–7.7)
Pain	1.0	4.7 (2.1–10.8)
Nausea/vomiting	1.0	3.0 (1.3–7.1)
Symptom items		
Dyspnea	1.0	1.3 (0.6–3.0)
Insomnia	1.0	8.2 (3.5–18.8)
Appetite loss	1.0	2.4 (1.0–5.5)
Constipation	1.0	4.4 (1.2–16.3)
Diarrhea	1.0	2.8 (1.1–7.1)
Financial difficulties	1.0	11.1 (3.4–36.5)

Adjusted for age, sex, comorbidity, psychiatric diagnosis, body mass index, smoking habits, alcohol intake, type of surgery, tumor stage, neoadjuvant therapy, and postoperative complications

## Discussion

Patients who suffered from reflux after oesophageal cancer surgery were more likely to experience sleep disturbances and poor sleep quality compared to patients without reflux. A combination of having reflux and sleep problems indicates a higher risk of reduced global quality of life, functional limitations, and more symptoms 1 year after oesophageal cancer surgery.

Previous literature indicates that there is a relationship between reflux and sleep disturbances in patients with gastro-oesophageal reflux disease [11, 26]. Similar results were seen for esophagectomy patients in this study. Few studies have addressed the problem of reflux and insomnia for this patient group. One study showed that sleep problems were common during the first postoperative days of surgery, and that sleep disturbances at diagnosis were predictive of sleep disturbances in the postoperative period [27]. In a more long-term perspective, sleep disturbances have been described to increase 1 year after minimally invasive esophagectomy [28]. These results are contradictory with findings in a systematic review, where insomnia was consistent over time [29]. In most studies, insomnia was not brought up as an important finding [6, 7, 30]. Sleep disturbances in cancer patients are a well-known [31, 32], but often neglected problem [33]. Sleep is essential for promoting healing, preventing risk of cancer recurrence, and improving cognitive functioning [32] and sleep disturbances have previously been reported to have a negative impact on patients’ HRQL [34, 35].

Our results favor the hypothesis that patients who have reflux after esophagectomy for cancer are more likely to suffer from sleep disturbances and poor HRQL. Because of the observational design of the study, strict causality cannot be implied. However, there was a consistent association between reflux and sleep problems, which was preserved after adjustment for possible important confounders, and argues for a valid conclusion. Patients who reported sleep disturbances also reported high risk of financial difficulties, and poorer role, emotional, and social function. This might imply that return to work for the survivors is affected, which in turn, could contribute to the worse global quality of life seen in this population. This warrants further investigation in future studies.

Since the problem with reflux increases when being supine, many patients reported that they slept with an elevated headrest. However, this did not seem to be sufficient for preventing sleep problems, nor did a combination of antireflux medication and an elevated headrest. This probably reflects the difficulty in alleviating nightly reflux. A previous study failed to show any long-term effects of surgical reflux prevention with cervical anastomosis, intrathoracic antireflux anastomosis, or pyloric drainage at 6 months or 3 years after esophagectomy [36]. In the future, studies about methods for alleviating reflux after oesophageal cancer surgery are warranted, especially since this common symptom reduces the patient’s wellbeing. Moreover, priority should be given to the assessment of sleep in cancer patients. The use of patient-reported outcomes with predefined cut-offs for symptom interventions in clinical practice has been shown to significantly improve patients’ survival and increase HRQL [37–39]. For curative intent oesophageal cancer patients, many symptoms may go undetected and untreated if not addressed by clinicians. Routine use of patient-reported outcome measures has been shown to improve communication about symptoms between patients and clinicians [40, 41]. One way of detecting burdensome symptoms is to let patients complete patient-reported outcome questionnaires in the waiting room and review the questionnaires during the clinical visit. Another option to guide symptom management is to monitor symptoms from home by asking patients to complete digital questionnaires between, for example, chemotherapy sessions.

Also, clinicians should inform patients about these common post-surgery problems and provide advice about how to optimally manage reflux and sleep disturbances. To date, few recommendations about how to manage sleep problems associated with reflux after oesophageal cancer surgery are available. This is still a rather unexplored area and until more evidence is available, clinicians may provide oesophageal cancer survivors with similar advice about how to manage these symptoms as given to patients with gastro-oesophageal reflux disease.

Strengths of this study include the prospective and population-based design, which counteracts potential selection bias. The use of validated questionnaires reduces information bias, but bias due to self-reporting could be present [42]. In our study, social desirability bias might be present since a research nurse conducted the data collection. However, misclassification would be non-differential between the exposure groups and therefore only dilute and not explain the associations. Reflux was self-assessed and this could introduce bias, but subjective assessment of reflux is currently regarded as the Gold Standard [43]. The definition of insomnia symptoms met to a high degree the criteria for insomnia in DSM-V [19]. Recall bias could be another problem with self-reported data. In this study, the recall period of reflux and HRQL was only 1 week, but for sleep assessment, the recall period was 3 months, which might increase the risk of recall bias. Moreover, the lack of pre-operative data on reflux and sleeping patterns prevents causal interference. The limited sample size, especially in the stratified analyses, which rendered wide confidence intervals, may also be a concern since a larger population would have contributed to more precise estimates. Confounding was mitigated by adjusting for several covariates in the analyses, but the risk of residual confounding cannot be eliminated.

In conclusion, this study suggests that patients with severe symptoms of reflux after oesophageal resection are more likely to suffer from sleep disturbances and poor sleep quality, which in turn are associated with HRQL reductions.
